# Identification of Novel Regulators of Radiosensitivity Using High-Throughput Genetic Screening

**DOI:** 10.3390/ijms23158774

**Published:** 2022-08-07

**Authors:** Rosette N. Tamaddondoust, Alicia Wong, Megha Chandrashekhar, Edouard I. Azzam, Tommy Alain, Yi Wang

**Affiliations:** 1 Radiobiology and Health, Canadian Nuclear Laboratories, Chalk River, ON K0J 1J0, Canada; 2 Department of Biochemistry, Microbiology and Immunology, University of Ottawa, Ottawa, ON K1N 6N5, Canada; 3 Molecular Biomedicine Program, Children’s Hospital of Eastern Ontario Research Institute, Ottawa, ON K1H 8L1, Canada

**Keywords:** radiosensitivity, radioresistance, genome editing, CRISPR-Cas9, ionizing radiation

## Abstract

The biological impact of ionizing radiation (IR) on humans depends not only on the physical properties and absorbed dose of radiation but also on the unique susceptibility of the exposed individual. A critical target of IR is DNA, and the DNA damage response is a safeguard mechanism for maintaining genomic integrity in response to the induced cellular stress. Unrepaired DNA lesions lead to various mutations, contributing to adverse health effects. Cellular sensitivity to IR is highly correlated with the ability of cells to repair DNA lesions, in particular coding sequences of genes that affect that process and of others that contribute to preserving genomic integrity. However, accurate profiling of the molecular events underlying individual sensitivity requires techniques with sensitive readouts. Here we summarize recent studies that have used whole-genome analysis and identified genes that impact individual radiosensitivity. Whereas microarray and RNA-seq provide a snapshot of the transcriptome, RNA interference (RNAi) and CRISPR-Cas9 techniques are powerful tools that enable modulation of gene expression and characterizing the function of specific genes involved in radiosensitivity or radioresistance. Notably, CRISPR-Cas9 has altered the landscape of genome-editing technology with its increased readiness, precision, and sensitivity. Identifying critical regulators of cellular radiosensitivity would help tailor regimens that enhance the efficacy of therapeutic treatments and fast-track prediction of clinical outcomes. It would also contribute to occupational protection based on average individual sensitivity, as well as the formulation of countermeasures to the harmful effects of radiation.

## 1. Introduction

During their lifetime, humans are likely to be exposed to various sources and doses of ionizing radiation (IR), whether from diagnostic examinations (e.g., computed tomography and nuclear medicine scans), environmental and occupational exposures, or therapeutic treatments of cancer and other diseases. Whether exposed to low-dose or high-dose ionizing radiation (LDIR, HDIR), DNA is the primary cellular target of IR. The DNA damage response is a safeguard mechanism that maintains genomic integrity in response to various forms of cellular stress, including IR [1]. If left unrepaired, DNA damage, particularly DNA double-strand breaks (DSBs) induced by IR, may lead to genomic instability resulting in homeostatic perturbations and detrimental consequences that are propagated to progeny cells. Whereas radiation dose and dose rate, along with genetic susceptibility and environmental factors, are known to determine the nature and magnitude of the cellular responses, the role of signaling pathways (e.g., in DNA repair, oxidative metabolism, or immune responses) remains unclear and is under investigation [2,3,4,5,6,7]. Identifying the molecular events involved in these pathways will shed light on novel biomarkers in key pathways that determine radiosensitivity. This review summarizes current findings, using high-throughput screening technologies, in identifying critical radiation resistance or sensitivity regulators in both normal tissue and cancer cells that could be used as therapeutic targets and also facilitate personalized treatment strategies.

## 2. The Radiation Response

Various responses to IR have been reported since Roentgen discovered X-rays in 1895. By 1906, the difference in radiosensitivities of the patients was noted as one of the major factors in influencing the outcome of radiotherapeutic treatments with X-rays medical application [8]. The definition of radiosensitivity, however, has been challenged in recent years. The Independent Advisory Group on Ionizing Radiation has redefined radiosensitivity as a measure of the degree of the cellular or organism response instead of a measure of IR-induced cell death [9]. In addition, “radioresistance” is a complex process in which multiple genes are involved in various mechanisms that prevent damage from occurring or that repair or eliminate damaged cells. The induced radioresistance of cancer cells or normal tissues also helps the cells to adapt to subsequent environmental challenges (i.e., IR), as well as to counteract harmful effects from oxidative metabolism [10].

The human response to IR is influenced by various factors such as age, smoking, diseases, and genotype [11,12]. For instance, an initial study in breast cancer patients evaluated that 81% to 90% of the variation in radiotherapy (RT)-induced normal tissue damage is due to patient-specific characteristics [13]. Such variation in radiosensitivity is partially influenced by an individual’s genetic or epigenetic profiles [12]. Although radiation-induced DNA damage can have different forms (i.e., base modifications, single-strand breaks, and DSB), radiosensitivity is the cellular capacity to perform specifically DNA DSB repair [14]. The two main pathways of DNA DSB repairs are error-prone non-homologous end joining (NHEJ), which is activated through the cell cycle, and homologous recombination repair (HRR), which occurs during the late S and G2 phases. The molecular events mediating these pathways continue to be understood and offer opportunities for novel discoveries [15,16]. Individual differences in the cellular capability of DNA repair mechanisms within human populations have been investigated mainly in the context of HDIR [11,14]. For instance, mutated *BRCA1/2* gene carriers experience greater radiosensitivity in both normal and tumor cells [17,18]. Moreover, frequent exposure to diagnostic radiation could be problematic, especially for younger individuals with adverse health effects manifesting at an older age. Exposure to cumulative doses of X-rays or CT scans enhances the risk of leukemia or brain cancer in children significantly [19]. However, the exact molecular basis of individual radiosensitivity, particularly in LDIR, remains poorly understood and the biomarkers of radiation sensitivity are elusive. With the large number (more than 20k) of genes in humans, low-throughput studies may not be efficient in screening all of the regulators involved in radiosensitivity. In contrast, high-throughput analyses provide for fast-tracking predictive testing and for tailoring therapeutic regimens or public policies for high-risk people in the event of radiation exposure.

## 3. High-Throughput Screening Methods to Study Radiosensitivity and Resistance

### 3.1. Gene Expression Analysis (Microarray and RNA-Sequencing)

**Figure 1:**
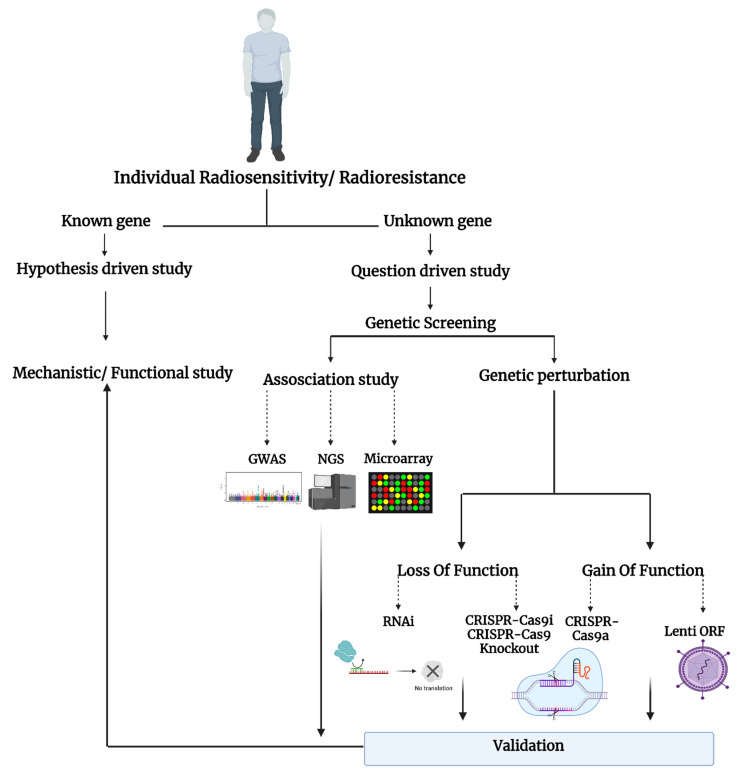
Schematic representation of the studies on individual radiosensitivity.

The question-driven (hypothesis-generating) high-throughput genetic screening studies, set to explore the unknown in an unbiased manner, have been more prominent with the development of critical “omics”–related technologies such as RNA-sequencing, microarray, RNAi, and CRISPR-Cas9. The high-throughput screening can identify novel genes or regulators of specific phenotypes and generate novel hypotheses that can be validated in low-throughput mechanistic and functional studies (Figure 1).

Using high-throughput gene expression methods, several studies have identified genes that influence the response to radiation. For instance, using DNA microarray analysis in lung cancer cells, Guo et al. profiled global gene expression in response to IR [20]. A microarray contains thousands of engineered complementary DNA (cDNA) oligonucleotides known as probes that hybridize with specific fluorescently labeled RNA molecules, and the expression of different known transcripts can be detected simultaneously [21]. Guo et al. focused their analyses on the expression of 143 genes in 2 lung cancer cell lines (NCI-H446 cells versus A549 cells) with different radiosensitivities in response to a single 5 Gy dose of gamma rays [20]. Compared to radiosensitive NCI-H446 cells, the expression of *XRCC5, ERCC5, ERCC1, RAD9A, ERCC4*, and *MDM2,* genes involved in DNA repair mechanism, was significantly increased in the radioresistant A549 cell line. The authors suggested this list of genes may prove useful in attempts to sensitize radioresistant lung neoplasms [22].

Performing next-generation RNA-sequencing (RNA-seq), extensive studies have been done on gene expression alterations in response to IR in a cell population as a whole. In a search for a predictor of response to IR in cancer cells, Young et al. took an RNA-seq approach to analyze the gene expression in radiosensitive LNCaP and radioresistant PC-3 prostate cancer cells [23]. They identified two canonical pathways with opposing responses in both cell lines 24 h after irradiation with high energy X rays: the DNA repair pathway (downregulation of *BRCA1*, *RAD51*, and *FANCG* in LNCaP and opposite pattern in PC-3 cell) and the cell cycle control of DNA replication pathway (downregulation of *ORC1*, *CDC6* and the *MCM* genes with contrasting pattern in PC-3 cell). In another study, the global gene expression in human glioma cells was assayed after exposure to a dose of gamma-rays leading to growth arrest. It was revealed that the inactivation of proapoptotic signaling molecules and late activation of antiapoptotic genes might contribute to the radioresistance of gliomas [24]. Deep sequencing was utilized to delineate different layers in the transcriptional response to IR in human breast cancer cells. This study identified protein-coding and previously unidentified non-coding genes that were responsive to IR [25]. Thus, RNA-seq allows for the complete sequencing of the whole transcriptome while microarray only profiles predefined transcripts through probe hybridization. In RNA-seq, purified RNA from genes and gene variants (e.g., splicing isoforms) are sequenced directly (without the help of the probes) [21]. Therefore, whereas both microarray and RNA-seq can show large numbers of differentially expressed genes, RNA-seq reveals an unbiased screening of a broader range of gene expression with higher specificity and sensitivity, including novel, coding, and non-coding transcripts, compared to microarrays [26].

Bulk RNA-seq analysis described in the previous section conventionally measures transcripts in a mixture of cells which allows the measurement of only the average transcript expression in a cell population. Such traditional sequencing methods are unable to analyze a small number of cells found in rare populations and also lose cellular heterogeneity information. Single-cell RNA-sequencing (scRNA-seq) is an innovative NGS approach that has enabled the measurement of the whole transcriptome at a single-cell resolution and contributed to understanding changes in the transcriptional circuitry of individual cells within their natural microenvironment. A scRNA-seq method was used in two different studies to investigate the acquired radioresistance in esophageal squamous cell carcinoma cells (ESCC). These studies revealed the cellular heterogeneity and dynamic gene expression changes in irradiated ESCC cells along with the genes and signaling pathways related to the development of radioresistance [27,28]. Similarly, scRNA-seq of breast cancer cell line MDA-MB-231 with and without IR treatment using the barcoded Smart-seq2 technology revealed a heterogeneous cellular response to DNA damage induced by IR. scRNA-seq data analysis also identified potential biomarkers of radiation sensitivity including *MCM3*, *MCM4* and *SLBP* genes involved in DNA replication [29]. Thus, single-cell sequencing technology has the power to delineate the heterogeneous response to IR in different cancer types and thereby improve treatment options.

However, although these platforms have helped identify numerous genes involved in radiosensitivity, the exact mechanism is still unclear. To understand the mechanism, a first and foremost step would be detecting the exact genome variant in a population [30]. Knowing the exact location would allow exploration of the transcription factor binding sites and affected regulatory factors [30]. However, this is challenging when the phenotype is influenced by more than one gene (polygenic pattern), in contrast to the Mendelian model where disease is caused by mutations in single genes on either the autosomes or sex chromosomes [31]. Radiosensitivity is a quantitative polygenic trait that is the product of interactions between cellular pathways [32]. For this reason, it would be appropriate to use genome-wide association studies (GWAS) that have successfully mapped thousands of loci and DNA sequence variations associated with complex traits underlying the risk of disease [31].

### 3.2. Genome-Wide Association Study (GWAS)

GWAS examines variations that are presented in the form of single nucleotide mutation. When the frequency of these mutations is more than 1% of the population, they are called single nucleotide polymorphisms (SNPs) [33]. In one of the first clinical studies of SNPs, Kerns et al. used the GWAS method to investigate genetic variants associated with erectile dysfunction as an indicator of normal tissue damage experienced after radiation therapy (RT) in prostate cancer patients [34]. From the high-throughput analysis of 512,497 SNPs, rs2268363, which is located in a gene whose encoded product affects male gonad development and function (the *FSHR* gene), was strongly associated with the development of long-term side effects of RT. This strongly supports the feasibility of using the GWAS approach in exploring the association between genetic predisposition and radiation injury in normal cells [34].

Moreover, although radiation-induced germline mutations or heritable genetic diseases in children of irradiated parents are still not confirmed, strong evidence of the heritability of the radiosensitivity trait in human somatic cells has been established [35,36]. In an attempt to discover genes and SNPs that affect radiosensitivity, Zyla et al. used genomic analysis from human twin pairs with the GWAS method and showed that about 66% of *CDKN1A* (cyclin-dependent kinase inhibitor 1A) expression in response to radiation is heritable [37]. *CDKN1A* encodes protein p21, a downstream effector of p53, and is one of the key regulators in cell cycle regulation and arrest following DNA damage. *CDKN1A* abnormal expression is associated with acute sensitivity to radiation. Moreover, GWAS allowed identification of SNPs that are significantly associated with *CDKN1A* expression (i.e., rs205543 (*ETV6* gene), rs2287505, and rs1263612 (*KLF7* gene) are involved in *CDKN1A* transcription factors, rs6974232 (*RPA3* gene), rs1133833 (*AKIP1* gene), and rs17362588 (*CCDC141* gene) are genes involved in DNA mismatch and RNA repair (summarized in Table 1) [37].

In addition, the *Drosophila melanogaster* Genetic Reference Panel (DGRP) is a valuable platform that allows GWAS and mapping analyses of potential genes, polymorphisms, or pathways influencing a particular quantitative trait [32]. Using this model, Vaisnav et al. discovered nine *Drosophila* genes (listed below and summarized in Table 1) with homologs in humans that are likely to be involved in radiation resistance [32]. Furthermore, the authors found 32 SNPs associated with radiation resistance (at *p* < 10^−5^, with two SNPs at *p* < 10^−6^). Among these novel candidates in radiation resistance, nine have human homologs with functions that are not actually involved in repair of DNA damage, highlighting the potential of the other mechanisms underlying radioresistance trait: human homolog proteins ATP5J (ATP synthesis), SLC family 35 member E1 (membrane transporter), coagulation factor II (blood coagulation), E3 ubiquitin ligase/SMURF2 (ubiquitination), protein VPRBP (cell cycle, telomerase regulation, and histone modification), transcription factor GATA-4 (embryogenesis, myocardial differentiation), dystonin/bullous pemphigoid antigen 1 (cell adhesion), LTrpC3/melastatin-2 (calcium signaling and homeostasis), and 5’-nucleotidase precursor (adenosine production) [32].

To construct a more precise and efficient polygenic risk model, Oh et al. used hundreds of SNPs and developed a machine learning algorithm called *pre-conditioned random forest regression* that signals even for small differential risks [47]. By applying this novel method to the GWAS cohort dataset of 368 prostate cancer patients treated with RT at a single institution, the team was able to identify the false positive SNPs and evaluated the importance of each SNP (the key biological function of each SNP) in inducing the radiotoxic outcomes [47]. However, the GWAS method comes with drawbacks that have been clearly discussed by Cano-Gamez et al. [31]. One major setback might be the lack of understanding of the roles of disease-associated loci in non-coding regions of the genome. As their role in gene expression regulation in different cell types or physiological contexts is still unclear, translating GWAS findings into clinical interventions might not be efficient [31]. Furthermore, the candidates found in GWAS or other methods discussed above need to be functionally validated. To achieve that, functional genomics techniques such as RNAi and CRISPR-Cas9 are powerful tools for analyzing gene function.

### 3.3. Genome-Wide RNAi Screening Method

RNA interference is a powerful method for loss-of-function genetic screens for key regulators and critical pathways involved in a particular phenotype [48]. This method has been used to knock down specific genes to investigate radiosensitivity of cancer cells [49]. For instance, using genome-wide RNAi screening to search for radioresistance genes in colorectal cancer cells (HCT116 and HCT15 cells), Wang et al. found that *RFC4* knockdown significantly mitigates X-ray-induced DNA damage repair and enhances apoptosis [38]. The protein encoded by the RFC4 gene facilitates cellular DNA DSB repair via a non-homologous end joining (NHEJ)-mediated pathway in colorectal cancer cells, and therefore RFC4 upregulation is associated with tumor progression (summarized in Table 1) [38]. In addition, five more genes, including NCAPH (regulatory subunit of the condensin complex), SYNE3 (transmission of mechanical forces across the nuclear envelope and in nuclear movement and positioning), *LDLRAD2* (receptor-mediated endocytosis), *NHP2* (required for ribosome biogenesis and telomere maintenance), and *FICD* (ATP binding activity) were also identified as potential candidate radioresistance genes.

Herr et al. used the same method to find homologous recombination repair (HRR)-specific factors in response to IR [39]. Since an intact sister chromatid template would be used in the HRR process, this pathway offers more accurate and error-free repair for DSBs (in comparison to the NHEJ pathway) [50]. The authors identified CDC73, a protein encoded by the *HRPT2* tumor suppressor gene, as a new regulator of HRR. By interacting with core histones of H2B and H3, CDC73 optimizes chromatin remodeling around DSBs and supports the accessibility of the DNA for downstream repair elements and events (summarized in Table 1) [39]. Van Haaften et al. exposed nematode *Caenorhabditis elegans* to 60 Gy of radiation and used a genome-wide RNAi technique to identify eight genes necessary to protect the germline against DNA DSB. Intriguingly, most of these newly identified genes with known human orthologs (i.e., *Y65B4BR.4A* (human: *WWP2*)*, H19NO7.2a* (human: *USP7, HAUSP*), *Y41C4a.10* (human: *TCEB2*)*, Y67D8C.5* (human: *UREB1*, *LASU1*), and *C52D10.9* (human: *SKP1A*)) are expected to play a role in the targeted degradation of proteins via the ubiquitination function. RAD51, histones, CDC25A, and p53, all of which play a role in DSB response, are regulated by ubiquitination. This observation supports the idea that certain proteins activate or regulate the DSB response pathway by undergoing proteasomal activity (summarized in Table 1) [40]. Knockdown of these genes improved sensitivity to ionizing radiation and amplified chromosomal nondisjunction [40]. In another study, van Haaften et al. expanded their data by identifying more genes that are active agents in DNA damage response and RNA processing and trafficking that contribute to increased radiosensitivity of germ cells in *C. elegans*. In addition, the novel genes were found to be strongly conserved throughout animal evolution. Among genes with human homolog, *ATM*, *ITGA6*, *NIPBL*, *NOB1*, *CAND1/TIP120*, *WWP2*, and *TopBP1* have been observed (summarized in Table 1) [41].

Although RNAi is a robust tool for genome-wide screening through the downregulation of gene expression at the mRNA level regardless of the target gene copy numbers, its off-target effects are also inevitable [51]. In fact, suppression of gene expression by RNAi might not be efficient, which may result in only a partial knockdown [51]. Many of these shortcomings of RNAi are effectively addressed by CRISPR-mediated gene editing technology.

### 3.4. Genome-Wide CRISPR-Cas9 Screening Method

Adopted from the bacterial immune system, Clustered Regularly Interspaced Short Palindromic Repeats (CRISPR)-Cas-associated protein 9, known as CRISPR-Cas9, is a novel technology that has revolutionized genome editing and gene therapy [52]. The CRISPR-Cas9 system comprises two biological components: the RNA-guided DNA endonuclease, Cas9, and the chimeric single-guide RNA (sgRNA) [53,54]. The sgRNA is loaded onto Cas9 and directed to a 20 bp region on the DNA target via base pairing. For functional gene editing, the target DNA must immediately precede a 5’ NGG sequence (N is any nucleotide), referred to as a protospacer adjacent motif (PAM). Cas9 promotes genome editing by inducing a DSB at the target genomic locus by re-direction to its target region. The cellular machinery then repairs the DNA DSB via NHEJ or HRR pathways [54].

This technique has been applied to investigate the effect of several genes (e.g., Hsp70, osteopontin, and HIF-1/2α) as critical regulators in radioresistance or radiosensitivity traits in different cell lines [55,56,57]. To develop a comprehensive approach and investigate radioresistance regulatory factors in the colorectal cancer (CRC) cell (RKO, HCT116, and SW620), Yu et al. applied genome-scale CRISPR sgRNA library in negative selection screens to identify radioresistance candidate genes. They found that DNA polymerase alpha 2 (POLA2), radical S-adenosyl methionine domain containing 2 (RSAD2), and microRNA5197-5p (miR-5197) had the most significant fold changes after IR exposure [45]. However, further investigation showed that overexpression of miR-5197 impaired radioresistance to a more considerable extent compared to other gene candidates. By inhibiting the expression of cell cycle regulatory protein CDK6 and promoting cell cycle arrest in the G1/S phase, miR-5197 contributes to IR-induced apoptosis in CRC (summarized in Table 1) [45]. The authors, however, emphasized the need for further studies with an in vivo model to prove their findings [45]. Using a genome-wide CRISPR-Cas9 sgRNA library for the first time in nasopharyngeal carcinoma (NPC) cells and performing high-throughput sequencing on sgRNAs obtained in a negative screen, Ziyan et al. found nine genes involved in the radiosensitivity or radioresistance of NPC cells [43]. Five genes (*BLN5*, *FAM3C, MUS81, DNAJC17, and CALD1*) were suggested as radiosensitivity modulators, whereas four genes (*CDKN2AIP, SP1, TOMM20*, and *SNX22*) seemed to be potentially radioresistant genes (summarized in Table 1). Additionally, an enrichment analysis of the KEGG database showed that these genes contribute to radiosensitivity or radioresistance in NPC via the Fanconi anemia pathway and TGF-beta signaling pathway. Through CRISPR/Cas9 high-throughput screening and negative selection of crucial genes that might be linked to radioresistance in NPC, Shen et al. also demonstrated that overexpression of LUC7L2 contributes to radioresistance via the autophagy process. LUC7L2 is an RNA binding protein that has not been fully studied and only has been characterized in recent years [58].

Hayman et al. performed a whole-genome CRISPR-Cas9 screen in an HNSCC cell line using treatment with ionizing radiation as a positive selection pressure to identify regulators of radiation sensitivity. Positive screening and NGS of sgRNAs enriched after multiple rounds of irradiation showed that activation of stimulator or interferon genes (known as STING, a signaling molecule associated with the endoplasmic reticulum) influences radiation response in HNSCC cells [44]. They further show that pharmacological activation of STING enhances the effects of ionizing radiation in vivo and might be a promising approach to enhance radiotherapeutic response in patients suffering from HNSCC (summarized in Table 1). In an interesting study, Zhu et al. performed genome-wide CRISPR activation screening and identified calcium-regulated heat-stable protein 1 (CARHSP1) as an essential element involved in radioresistance traits in human glioblastoma cells (summarized in Table 1) [42]. Because of its cold-shock domain, CARHSP1 has the capacity to bind to polypyrimidine regions of single-stranded RNA, single-stranded DNA, or double-stranded DNA [59]. Hence, CARHSP1 can bind to DNA and regulate the rate of transcription termination, but also it has the potential to regulate RNA stability, mRNA degradation, and ribosomal translation [60]. Intriguingly, CARHSP1 enhances mRNA stability of tumor necrosis factor-alpha (TNF-α), a crucial pleiotropic cytokine and a critical inflammatory molecule [60]. With this information, Zhu et al. showed that an elevated level of CARHSP1 is associated with radioresistance of glioblastoma cells via CARHSP1/TNF-α pathway signaling [42]. Cheng et al. used an unbiased genome-wide CRISPR/Cas9 knockout strategy in A549 lung cancer cells and identified plakophilin 2 (PKP2) as a critical driver of radiation resistance in lung cancer cells [61]. Cheng et al. have shown for the first time that methylated PKP2 protein promotes NHEJ and increases lung cancer radioresistance. Arginine methylation of PKP2 is mediated by protein arginine methyltransferase-1 (PRMT1). Hence, PRMT1 inhibition may also be an attractive approach to radiosensitize lung cancer [61].

Altogether, these studies show that the application of the CRISPR/Cas technique offers an unbiased global screen and a comprehensive map of the genes and pathways that are involved in IR-induced response.

## 4. Discussion

Gene expression profiling describes the simultaneous measurement of the expression of many genes or the entire genome. It can be accomplished by assessing mRNA levels with two major platforms: microarray and RNA-seq. Tracing transcripts profiles that are differentially expressed in different cell types maintained in different contexts (e.g., environments or stress factors) gives a map of the association between genotype and a particular phenotype. In contrast to microarray, where the expression of only known gene sequences can be assessed, RNA-seq offers a “de novo” readout where prior knowledge of the reference genome or sequence of interest is not available. However, for the phenotypes whose genome has a polygenic pattern, GWAS has zoomed into the nucleotide variation and SNPs to offer a precise readout of the DNA sequence associated with complex traits. Further, powerful genomic tools such as RNAi and CRISPR-Cas9 have enabled comprehensive analyses of gene function.

RNAi silences genes by knocking down the mRNA of a gene, whereas CRISPR generates gene knockouts by targeting the DNA sequence. Although RNAi-based screening has been helpful in deciphering the elements directing radiosensitivity of the cells, the utility of RNAi has been hindered by imperfect mRNA knockdown, confounding off-target effects (introducing noises); this makes interpretation of phenotypic changes difficult and limits the method to transcribed genes. Additionally, the introduction of RNAs may trigger immune responses. CRISPR-Cas9 technology has changed the landscape of gene-editing technology by effectively addressing many of these limitations by enabling targeted modification of DNA to achieve complete gene knockout. In addition, it provides an opportunity to introduce nucleotide variation and to compare and measure the chromosome damage after IR treatment in such edited cells, thereby identifying the nucleotide variation of interest that influences the radiosensitivity.

Both positive and negative screens in the genetic perturbation studies have been used in radiation research; however, the purposes and outcomes of the screens are different. Negative screens are used to find genes that cause radiation resistance, and positive screens are used to find genes that cause radiation sensitivity. In the negative screening approach, the CRISPR-edited cancer cells are treated with a sublethal dose of radiation (which may kill ~20% of cells). Compared to the control (CRISPR-edited but not treated with radiation) cells, sgRNAs targeting genes involved in mediating radiation resistance are depleted from the population over time. In the positive screens, CRISPR-edited cells are treated with a lethal dose of radiation such that sgRNAs targeting genes involved in mediating radiation sensitivity are enriched in the population over time and can be identified upon sequencing. Positive and negative screening can be performed simultaneously to identify regulators of radioresistance and radiosensitivity, respectively, with the use of a specific radiation dose. Moreover, CRISPR is a versatile tool and can perform not only loss-of-function but also gain-of-function screening. The gain-of-function application of CRISPR-Cas9 based on using a nuclease-null Cas9 protein (dCas9) fused to transcriptional activators enables a quick and efficient increase in target endogenous gene expression. Similarly, CRISPR inhibition can be performed by fusing the dCas9 to a transcriptional repressor. Both CRISPR inhibition and CRISPR activation libraries have been used in the radiation research field.

Genome-wide studies of radioresistance and radiosensitivity are all performed in two-dimensional (2D) cell cultures, which is different from the microenvironment of either tumor or normal tissues. For example, the 2D cancer cell culture model lacks critical features of tumor cells such as partial oxygen pressure, altered cell–cell contact, cell base membrane adhesion, and reprogrammed metabolism. Although functional genomics in 2D cell culture has produced a wealth of information and has uncovered novel regulators, they often failed to reflect the critical aspects in vivo. For example, in DepMap, a project to discover cancer drivers using genome-scale CRISPR screens in hundreds of cell lines, <1% of the top 1000 hits show a positive growth effect [62]. Moreover, even the inactivation of known tumor suppressor genes in cancer cells maintained in 2D architecture often leads to negative phenotypes. Therefore, genome-wide screening of cells grown in three-dimensional (3D) culture systems closely mimics the in vivo tumor microenvironment and is highly desirable. Han et al. investigated the genome-wide CRISPR screen in a 3D lung cancer spheroid model and discovered cancer cell sensitivities different from those of the monolayer 2D culture. Since the 3D spheroid model more accurately recapitulates the microenvironment of in vivo tumors, Han et al. took advantage of this model while using the CRISPR screen (summarized in Table 1) [46]. Moreover, taking advantage of the combination of CRISPR-Cas9 and 3D cell spheroid culture, Lan et al. detected DYRK1A as a sensitive target for radiotherapy in pancreatic cancer cells [63]. Alternately, genome-wide screening to identify essential genes can be done under different oxygen and metabolic conditions (such as hypoxia or low glucose) that match physiological conditions.

Furthermore, in order to obtain a comprehensive map of radiosensitizing and radioresistance genes, integration of the genetic, transcription, and translational datasets in response to various radiation doses have to be integrated. Whereas some researchers have shown that radiation-induced gene expression is highly dependent on the individual cell genotype [64,65], evidence shows that when evaluated at the level of translation, radiation-induced gene expression is significantly associated with tissue-type dependency [66]. Stackhouse et al. generated glioblastoma (GBM) patient-derived xenograft (PDX) models and applied a novel bioinformatics pipeline to analyze phenotypic, transcriptomic, and global kinomic (functional proteomic) profiles [67]. Conducting whole-exome sequencing (WES) and deep RNA-seq, the authors suggested that phenotypic changes such as radiotherapy resistance are not mediated only at the genomic level, but instead, largely at the epigenetic, post-transcriptomic, and post-translational level [67].

Moreover, genetic screening studies conducted so far have been using HDIR to contribute to enhancing radiation therapy effectiveness, for example, how to increase the radiosensitivity of cancer tissue. This is in contrast to the radiosensitivity to LDIR to which one is often exposed. Also, LDIR does not have the selection pressure that HDIR does, and the exact cellular stress-induced mechanisms and pathways in response to LDIR are not well understood. To find a precise reporter (e.g., DNA repair reporter) in the context of LDIR, CRISPR-Cas9 can also be a powerful tool that enables comprehensive analyses of critical molecules and pathways activated following LDIR exposures.

Recent advances in genome editing have enabled editing SNPs without breaking DNA, opening new avenues to study genetic variants associated with particular phenotypes or diseases. For instance, the new generation of base editors, adenine base editors (ABEs), enable the direct mutation at target loci in living cells without activating DSB damage response [68]. This method has optimized the conversion of unwanted alleles into nan-pathogenic alleles and enabled phenotypic rescue with minimum genotoxic effects [69]. With prime editor systems, such as prime editing guides (pegRNAs), any local mutation and desired edit (up to dozens of base pairs) can be performed at the target site [70]. Some pitfalls of CRISPR’s genetic scissors technique, including uncontrolled mixtures of editing outcomes, p53 activation, and larger DNA rearrangement, can be avoided with these methods, making them safe and precise approaches in the context of radioprotection and radiosensitivity studies.

Taken all together, by expanding our understanding of radiogenetics and the mechanism involved in cellular radiosensitivity, we can identify genes that can predict clinical outcomes. With such prediction, alternate treatments could be considered for patients prone to hyper-radiosensitivity [71]. Moreover, if a considerable variation of risk is identified in a particular population subgroup, a more tailored protection system can be proposed to protect specific individuals.

## Figures and Tables

**Table 1:** Summary of selected current research on radiation resistance.

Authors	Method/Dose Type	Model/Cell Type	Findings
Wang et al. [38]	Genome-wide RNAi screen/ Single dose 6 Gy, X-ray	Colorectal cancer cells exposed to X-rays both in vitro and in a mouse model	RFC4 protects colorectal cancer cells from radiation-induced DSBs and apoptosis both in vitro and in vivo; RFC4 enhances radioresistance.
Herr et al. [39]	Genome-wide RNAi screen/ Single dose 4 Gy (1.96 Gy min^−1^, Cs^137^)	Human bone osteosarcoma epithelial cells (U2OS line)	CDC73 is an important regulator of HRR-mediated DNA repair and genome stability. CDC73 enhances radioresistance.
van Haaften et al. [40]	Genome-wide RNAi screen/ Single dose 60 Gy, Gammacell 1000 (Cs-137)	*C. elegans* strains: wild-type Bristol N2, NL1832 (*pk732*), and TY1774 *yIs2* [*xol-1*::*lacZ rol-6* (pRF4)] IV.	Genes involved in the cellular response to DNA DSBs were identified.
van Haaften et al. [41]	Genome-wide RNAi screen/Single dose 140 Gy a Gammacell 1000 (Cs-137)	*C. elegans* strains were used: wild-type Bristol N2, atm-1 (gk186), lig-4 (ok716), and cku-80 (rb964)	A total of 45 *C. elegans* genes were identified that increased sensitivity to ionizing radiation in germ cells.
Kerns et al. [34]	GWAS/ 39 to 42 fractions of 1.8 Gy Xray	DNA isolated from lymphocytes	The location of SNP that is associated with erectile dysfunction as a side effect of RT was identified. These SNPs are specific for only patients with African ancestry.
Zyla et al. [37]	GWAS/ Single dose of 2 Gy of X-ray (0.5 Gy/min)	Blood T lymphocytes	SNPs influencing radiation sensitivity were identified.
Vaisnav et al. [32]	GWAS/Continuous exposure (4 h and 45 min) of gamma rays, 4.85 Gy/min, resulting in a total dose of 1382 Gy	*Drosophila* Genetic Reference Panel (DGRP)	Novel genes associated with variation in radiation resistance were identified.
Zhu et al. [42]	Whole CRISPR-Cas9 screen (positive screen) Treated with dose rate of 12, 15 Gy/min with X-ray Irradiator for three rounds	Glioblastoma cells	CARHSP1 enhances radioresistance in glioblastoma cancer cells.
Ziyan et al. [43]	Whole CRISPR-Cas9 screen (negative screen)/ Single dose 2 Gy	Nasopharyngeal carcinoma	Nine genes involved in the radiosensitivity or radioresistance of NPC cells were identified.
Hayman et al. [44]	Whole CRISPR-Cas9 screen (positive screen)	Neck squamous carcinoma cells (HNSCC)	Knockout of STING significantly increases radiation survival in both in vitro and in vivo models.
Yu et al. [45]	Whole CRISPR-Cas9 screen (negative screen) 6 & 12 single doses of X rays; dose rate: 5 Gy/min	Colorectal cancer cells	By inhibiting expression of cell cycle regulatory protein CDK6 and promoting cell cycle arrest in G1/S phase, microRNA-5197-5p (miR-5197) was reported as a radiosensitization factor.
Han et al. [46]	Whole CRISPR-Cas9 screen (positive screen)	Non-small-cell lung carcinoma cell lines	Key differences between 2D monolayer and 3D spheroid cancer models in CRISPR screen was demonstrated.

## Data Availability

Not applicable.
